# Interruptions of the *FXN* GAA Repeat Tract Delay the Age at Onset of Friedreich’s Ataxia in a Location Dependent Manner

**DOI:** 10.3390/ijms22147507

**Published:** 2021-07-13

**Authors:** Suran Nethisinghe, Maheswaran Kesavan, Heather Ging, Robyn Labrum, James M. Polke, Saiful Islam, Hector Garcia-Moreno, Martina F. Callaghan, Francesca Cavalcanti, Mark A. Pook, Paola Giunti

**Affiliations:** 1 Ataxia Centre, Department of Clinical and Movement Neurosciences, UCL Queen Square Institute of Neurology, Queen Square, London WC1N 3BG, UK; s.nethisinghe@ucl.ac.uk (S.N.); maheswaran.kesavan.18@alumni.ucl.ac.uk (M.K.); heather.ging.14@alumni.ucl.ac.uk (H.G.); h.garcia-moreno@ucl.ac.uk (H.G.-M.); 2 Neurogenetics Service, Rare and Inherited Disease Laboratory, London North Genomic Laboratory Hub, Great Ormond Street Hospital for Children NHS Foundation Trust, London WC1N 3BH, UK; r.labrum@nhs.net (R.L.); james.polke@nhs.net (J.M.P.); 3 UCL Queen Square Institute of Neurology, London WC1N 3BG, UK; afm.islam@ucl.ac.uk; 4 Wellcome Centre for Human Neuroimaging, UCL Queen Square Institute of Neurology, University College London, London WC1N 3AR, UK; m.callaghan@ucl.ac.uk; 5 Institute for Biomedical Research and Innovation (IRIB), Italian National Research Council (CNR), 87050 Mangone, Italy; francesca.cavalcanti@irib.cnr.it; 6 Ataxia Research Group, Division of Biosciences, Department of Life Sciences, College of Health and Life Sciences, Brunel University London, Uxbridge UB8 3PH, UK; markpook@me.com; 7 Synthetic Biology Theme, Institute of Environment, Health and Societies, Brunel University London, Uxbridge UB8 3PH, UK

**Keywords:** Friedreich’s ataxia, FRDA, ataxia, GAA repeat interruption, triplet repeat primed PCR, TP PCR, frataxin, *FXN*

## Abstract

Friedreich’s ataxia (FRDA) is a comparatively rare autosomal recessive neurological disorder primarily caused by the homozygous expansion of a GAA trinucleotide repeat in intron 1 of the *FXN* gene. The repeat expansion causes gene silencing that results in deficiency of the frataxin protein leading to mitochondrial dysfunction, oxidative stress and cell death. The GAA repeat tract in some cases may be impure with sequence variations called interruptions. It has previously been observed that large interruptions of the GAA repeat tract, determined by abnormal *Mbo*II digestion, are very rare. Here we have used triplet repeat primed PCR (TP PCR) assays to identify small interruptions at the 5′ and 3′ ends of the GAA repeat tract through alterations in the electropherogram trace signal. We found that contrary to large interruptions, small interruptions are more common, with 3′ interruptions being most frequent. Based on detection of interruptions by TP PCR assay, the patient cohort (*n* = 101) was stratified into four groups: 5′ interruption, 3′ interruption, both 5′ and 3′ interruptions or lacking interruption. Those patients with 3′ interruptions were associated with shorter GAA1 repeat tracts and later ages at disease onset. The age at disease onset was modelled by a group-specific exponential decay model. Based on this modelling, a 3′ interruption is predicted to delay disease onset by approximately 9 years relative to those lacking 5′ and 3′ interruptions. This highlights the key role of interruptions at the 3′ end of the GAA repeat tract in modulating the disease phenotype and its impact on prognosis for the patient.

## 1. Introduction

Friedreich’s ataxia (FRDA) is a comparatively rare autosomal recessive disease primarily caused by the homozygous expansion of a GAA trinucleotide repeat in intron 1 of the *FXN* gene. It is one of the most common inherited ataxias with a prevalence of 1.8 per 100,000 in the UK [1]. It is characterised by neurological features such as loss of coordination and balance as well as dysarthria, weakness and deep sensory loss, whilst non-neurological signs include hypertrophic cardiomyopathy, diabetes myelitis, kyphoscoliosis and foot deformities [2,3,4,5]. The onset of symptoms usually occurs before the age of 20 years, with most cases developing by 25 years [3,6].

In the general population, the *FXN* GAA repeat tract contains 5–68 repeats, whereas fully-penetrant expansions can range from 66 to 1700 GAA repeats, with the majority typically between 600 and 1200 repeats [6,7,8,9,10,11,12]. However, an affected individual has been reported with a 56 GAA repeat allele [13]. A total of 96% of FRDA patients are homozygous for GAA expansions whilst the remaining patients are compound heterozygous for a GAA repeat expansion and a second *FXN* mutation [10,14,15]. Age at disease onset decreases with increasing GAA repeat length, particularly for the shorter allele (GAA1) [6,11], with disease onset previously predicted to occur 2.3 years earlier for every 100 GAA repeats added to GAA1 [2].

The GAA repeat size accounts for only about 36–56% of the variation in age of onset [2,6,11]. This suggests that there are other mechanisms contributing to this variation including, but not limited to, somatic mosaicism, other modifying genes, environmental factors or interruptions in the GAA repeat [2,6,11].

Due to the length of the majority of expanded alleles found in FRDA patients, sequencing of the GAA repeat tract has been limited. Studies where relatively short expansions, up to about 130 repeats, have been sequenced show that the GAA repeat tract can be interrupted with sequences such as (GAGGAA)_5–9_ or (GAAGGA)_65_ and these are associated with either the absence of the FRDA disease phenotype [7,8,16] or atypical mild late-onset or very late-onset FRDA disease [7,9,12,17,18,19]. Therefore, interruptions may stabilise the expansion of the repeat tract as observed through the quite stable transmission of a 112 repeat tract containing a (GAAAGAA)_2_ interruption through two generations [7]. Interrupted (ATTCT)_n_ repeats have also been shown to modify the phenotype in spinocerebellar ataxia type 10 (SCA10) [20], whilst we have previously shown that interruptions in the spinocerebellar ataxia type 1 (SCA1) pathogenic (CAG)_n_ repeat alleviate the disease phenotype [21].

Interestingly, one study found that interruptions were clustered at the 3′-ends of the expanded repeats, affecting the last 10–15 triplets [22]. Indeed, the (GAAAGAA)_2_ interruption observed in a stably transmitted 112 repeat tract was located about 20 repeats from the 3′ end of the tract [7]. It should be noted that these interruptions revolve around single nucleotide point mutations, insertions or deletions of the basal GAA repeat tract. Restriction enzyme digestion can be used to identify the presence of specific interruptions, such as *Ear*I and *Mnl*I [22], whose recognition sequences are GAAGAG and GAGG respectively, or absence of interruptions with *Mbo*II [23,24], whose recognition sequence of GAAGA permits the digestion of uninterrupted GAA repeat stretches. The latter can identify non-specific interruptions and has been used to show that large interruptions in the FRDA GAA repeat tract are very rare, with the vast majority (97.8%) of patient samples lacking significant sequence changes that would alter their *Mbo*II digestion profiles [24]. In that study small interruptions were found at the 3′ end of the repeat tract in three out of the nine FRDA samples sequenced, with sequences […(GAA)_23_A(GAA)_5_AGAA], […(GAA)_26_A(GAA)_4_A(GAA)_2_] and […(GAA)_4_GAG(GAA)_5_] [24].

Here we screened a cohort of 101 patients with FRDA for interruptions at the 5′ and 3′ ends of the *FXN* GAA repeat tract using a triplet repeat primed PCR (TP PCR) assay. Small interruptions detected by this method are more common in our cohort than the large interruptions previously reported [24]. Furthermore, interruptions at the 3′ end of the GAA repeat tract are associated with shorter GAA1 repeat tracts and a later age at disease onset. The decrease in age at disease onset with increasing number of GAA1 repeats was modelled as an exponential decrease that depended on the location of interruptions within the GAA repeat tract (absent, 5′ end, 3′ end or both 5′ and 3′ ends). The importance of accounting for interruptions, and their specific type, is evidenced by the fact that not doing so would lead to the predicted age at disease onset for the 3′ interruption subgroup being, on average, 9 years earlier than observed clinically. This also serves to highlight the substantial impact interruptions at the 3′ end of the GAA repeat tract have in modulating the disease phenotype and determining the prognosis for the patient.

## 2. Results

### 2.1. GAA Repeat Tract Interruptions Can Be Detected through Alteration of TP PCR Electropherograms

**Figure 1:**
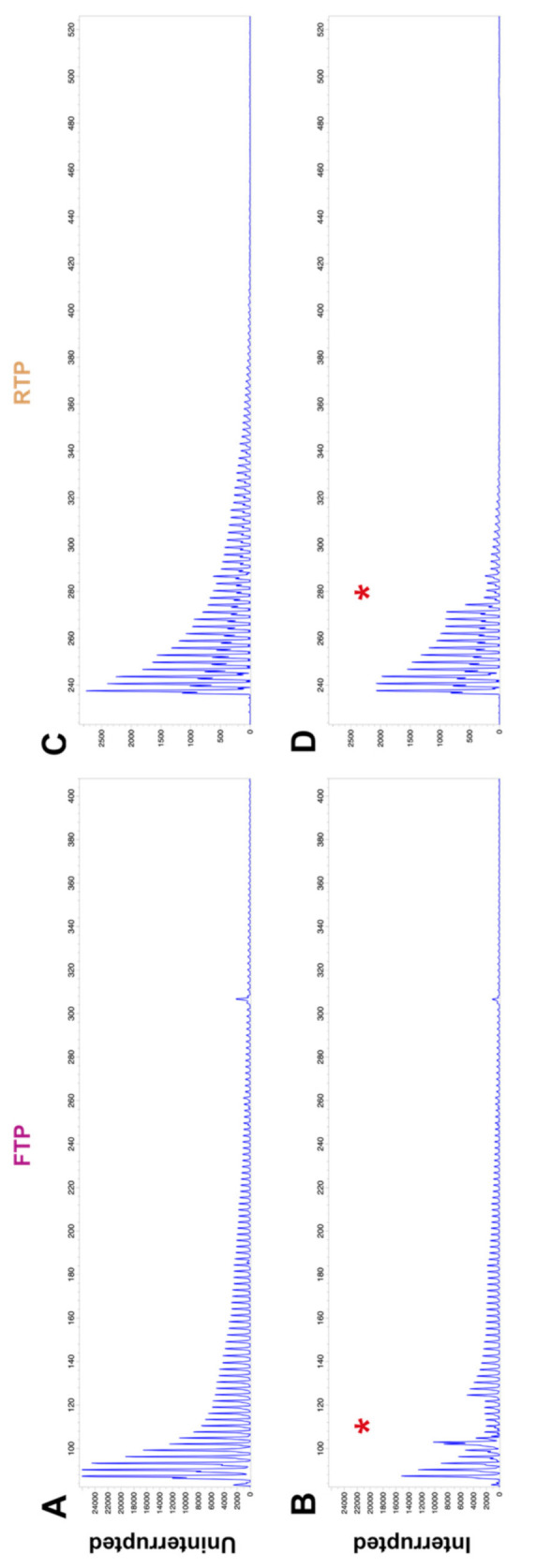
Example Forward and Reverse TP PCR electropherograms showing uninterrupted and interrupted repeat traces. (**A**) Uninterrupted FTP electropherogram for an individual with 450 and 720 GAA repeats. (**B**) FTP electropherogram for an individual with 200 and 1000 GAA repeats, showing a drop in signal indicating a 3′ interruption (*). (**C**) Uninterrupted RTP electropherogram for the same individual as shown in (**A**). (**D**) RTP electropherogram for an individual with 1100 and 1200 GAA repeats, showing a drop in signal indicating a 5′ interruption (*).

**Figure 2:**
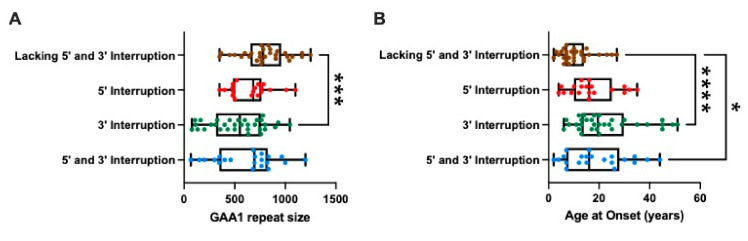
Interruption of the *FXN* GAA repeat tract is associated with shorter GAA1 repeat sizes and a later age at disease onset. (**A**) Box-and-whisker plot showing the distribution of the GAA1 repeat sizes for each interruption subgroup of the cohort. Kruskal–Wallis and subsequent Dunn’s multiple comparisons tests show that the 3′ interruption subgroup has significantly smaller GAA1 repeat sizes compared to the subgroup lacking 5′ and 3′ interruptions. Other subgroup comparisons were not significant. (**B**) Box-and-whisker plot showing the distribution of ages at disease onset across each interruption subgroup of the cohort. Kruskal–Wallis and subsequent Dunn’s multiple comparisons tests show that both the 3′ interruption subgroup and the 5′ and 3′ interruption subgroup have significantly later ages at disease onset compared to the subgroup lacking 5′ and 3′ interruptions. Other subgroup comparisons were not significant. The whiskers indicate the minimum and maximum values whilst the box shows the 25th to 75th percentiles of the data with a line indicating the median. * *p* ≤ 0.05; *** *p* ≤ 0.001; **** *p* ≤ 0.0001.

Previously, Forward TP PCR (FTP) had been developed as a diagnostic tool to detect the presence of a GAA repeat expansion in the intron 1 of the *FXN* gene [25]. The fundamental principle behind the method is the use of a repeat-specific primer which binds to the repeat at multiple sites leading to a mixture of products of varying size dependent on the size of the repeat tract [26]. Smaller repeats are amplified more frequently, giving rise to a characteristic electropherogram trace consisting of a ladder of peaks with a 3 bp periodicity that gradually diminishes in signal with increased product size (Figure 1A,C). The repeat-specific primer used in this assay binds to a stretch of seven GAA repeats. Interruptions of the GAA repeat tract sequence affect primer binding leading to a drop in signal, which is observed as a gap in the TP PCR electropherogram ladder (Figure 1B,D). In addition to the previously published FTP [25], which examines the 3′ end of the repeat tract, a Reverse TP PCR (RTP) assay was devised to examine the 5′ end of the repeat tract. Example FTP and RTP electropherograms, without and with interruptions, are shown in Figure 2. The FTP assay is only sensitive enough to detect interruptions located within about 100 repeats from the 3′ end of the repeat tract whilst the weaker signal for the RTP assay limits interruption detection to about 60 repeats from the 5′ end of the repeat tract. The cohort can be subdivided based on the presence and location of interruptions. Where interruptions could not be detected by TP PCR assay, small interruptions in the middle of the repeat could not be ruled out so this group is referred to as Lacking 5′ and 3′ interruption. The number of individuals in each subgroup of the cohort are shown in Table 1. Out of the cohort of 101, 72 individuals (71.3%) had an interruption at either end of the repeat tract. In total, 19 individuals in the cohort had only a 5′ interruption (18.8% of the cohort or 26.4% of individuals with interruptions) whilst 32 individuals only had a 3′ interruption (31.7% of the cohort or 44.4% of individuals with interruptions). A total of 21 individuals had interruptions at both 5′ and 3′ ends (20.8% of the cohort or 29.2% of individuals of interruptions).

### 2.2. Interruption at the 3′ End of the GAA Repeat Tract Is Associated with a Shorter GAA1 Repeat Size and a Later Age at Onset

The range of GAA1 repeat sizes differs between subgroups, with 3′ interruption and 5′ and 3′ double interruption groups having more individuals with GAA1 repeats less than 350 repeats compared to those lacking 5′ and 3′ interruption or 5′ interruption alone. The details of the cohort are summarised in Table 1.

To explore the impact of interruptions, the data were plotted in box-and-whisker diagrams based on either GAA1 repeat size (Figure 2A) or age at disease onset (Figure 2B). The Kruskal–Wallis and subsequent Dunn’s multiple comparisons tests showed that the 3′ interruption subgroup is comprised of individuals with significantly smaller GAA1 repeat sizes (adjusted *p* = 0.0004) than the group lacking 5′ and 3′ interruptions. Both the 3′ interruption (adjusted *p* < 0.0001) and the 5′ and 3′ interruption subgroups (adjusted *p* = 0.0281) had significantly later ages at disease onset than the subgroup lacking either 5′ or 3′ interruptions.

### 2.3. Modelling the Impact of Interruptions by Subgrouping

**Figure 3:**
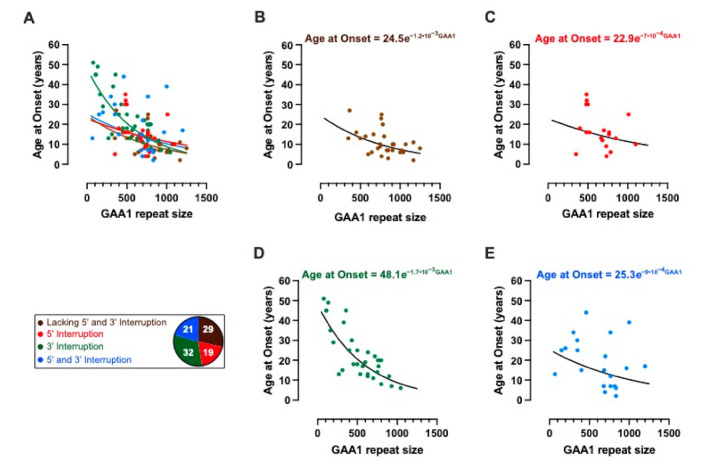
Ages at disease onset with respect to the smaller *FXN* GAA (GAA1) repeat size. The age at disease onset monotonically decreased with increasing GAA1 repeat size. This observed relationship was modelled as an exponential decrease, on a groupwise basis (adjusted *R*^2^ = 0.342; F-statistic = 8.43, *p* = 5.96 × 10^−8^). (**A**) Actual and modelled dependence of the age at disease onset on GAA1 repeat size for the whole FRDA cohort (*n* = 101). The subgroup membership is colour-coded according to the legend, which also indicates the number of patients per subgroup. (**B**–**E**) show the data, model results and model equation for each subgroup separately.

**Figure 4:**
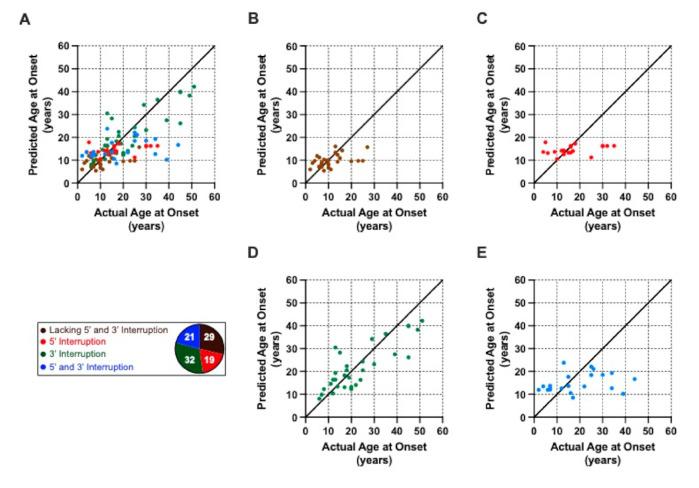
Predicted versus actual ages at disease onset for a given number of GAA1 repeats. (**A**) Actual and predicted ages at disease onset for the whole FRDA cohort (*n* = 101). The subgroup membership is colour-coded according to the legend, which also indicates the number of patients per subgroup. (**B**–**E**) show the predicted versus actual age at disease onset for each subgroup separately. In each graph, the solid black line indicates identical predicted and actual ages at disease onset.

We used the cohort data to fit a model to understand how interruptions influence the age at disease onset. The model fit resulted in an adjusted *R*^2^ of 0.342. The actual cohort data (dots) and the modelled dependence of the age at disease onset on GAA1 repeat size (lines) for the whole cohort is shown in Figure 3A, with individual interruption subgroups and their respective models shown in Figure 3B–E. The clinically observed and predicted ages at disease onset are shown for the whole cohort in Figure 4A, and for each separate interruption subgroup in Figure 4B–E.

**Figure 5:**
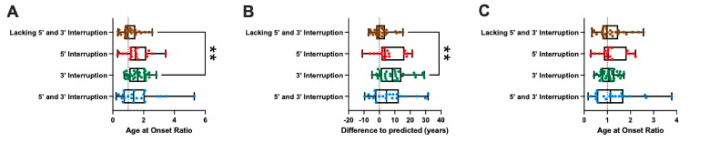
Interruption at the 3′ end of the *FXN* GAA repeat tract is associated with a delayed age at disease onset. (**A**) Box-and-whisker plot showing the age at onset ratio (Actual/Predicted) when using the model of the subgroup lacking interruptions to predict age at onset for all members of the cohort. Kruskal–Wallis and subsequent Dunn’s multiple comparisons tests show that the 3′ interruption subgroup had significantly greater age at onset ratios compared to the subgroup lacking 5′ and 3′ interruptions. (**B**) Box-and-whisker plot showing the differences in actual to predicted ages at onset for all members of the cohort when using the model lacking interruptions to predict age at onset. Kruskal–Wallis and subsequent Dunn’s multiple comparisons tests show that the 3′ interruption subgroup has significantly later ages at disease onset compared to the subgroup lacking 5′ and 3′ interruptions. Patients with a 3′ interruption present with FRDA approximately 9 years later than predicted on average compared to those lacking interruptions, based on the prediction model for individuals lacking 5′ and 3′ interruptions. (**C**) Box-and whisker plot showing the age at onset ratio when using subgroup-specific models, which shows that these models more accurately predict age at onset. There is no longer a significant difference between the 3′ interruption subgroup and that lacking 5′ and 3′ interruptions. The whiskers indicate the minimum and maximum values whilst the box shows the 25th to 75th percentiles of the data with a line indicating the median. The dotted line indicates an age at onset ratio of 1 or a difference to predicted age at onset of 0. ** *p* ≤ 0.01.

We then explored the suitability of group-specific modelling. The age at onset ratios for each interruption subgroup are shown in box-and-whisker plots (Figure 5A). The dotted line indicates a value of 1, such that the predicted age at onset matches the actual age at onset. The 3′ interruption group has a median age at onset ratio of 1.623 (1.622 ± 0.5544; mean ± S.D.) which is significantly greater than the subgroup lacking 5′ and 3′ interruptions (adjusted *p* = 0.0061). This corresponds to a later age at disease onset for individuals with a 3′ interruption than would be predicted if they did not have a 5′ or 3′ interruption. The differences between the actual and predicted ages at onset for each interruption subgroup are also shown in box-and-whisker diagrams (Figure 5B). The dotted line indicates a difference of zero, i.e., where the predicted age at onset matches the actual age at disease onset. The 3′ interruption subgroup had a significantly later age at disease onset compared to the subgroup lacking 5′ and 3′ interruptions (adjusted *p* = 0.0014). The 3′ interruption group has a median difference in age at onset of 8.5 years (9.4 ± 9.7 years; mean ± S.D.). This data indicates that patients with a 3′ interruption present with FRDA approximately 9 years later than would be predicted for those lacking 5′ and 3′ interruptions.

Importantly, no such differences were observed when the appropriate groupwise model was used (adjusted *p* > 0.9999).

## 3. Discussion

Previously, we have observed that significant sequence changes to the GAA repeat tract that would alter *Mbo*II digestion profiles are rare, suggesting that most FRDA patients have mainly pure GAA repeat expansion throughout most of the length of the repeat tract [24]. The main limitation of the *Mbo*II restriction digestion method is that it cannot detect small, localised repeat interruptions. Despite this, it was shown that a significant number of FRDA samples (3 out of 9), when sequenced, contained small sequence interruptions located at the 3′ end of the GAA repeat tract, which would not be detected by *Mbo*II restriction digestion [24]. Here we present our findings using TP PCR assays examining the presence of these small sequence interruptions at the 5′ or 3′ ends of the GAA repeat tract. We found that, contrary to the larger interruptions detected by *Mbo*II restriction digestion, smaller interruptions are very common with 71.3% of the 101 FRDA patients in our cohort having an interruption at either end of the repeat tract. In the subset of patients with interruptions, most have 3′ interruptions (44.4%), followed by those with both 5′ and 3′ interruptions (29.2%) and finally those with just 5′ interruptions (26.4%). This is in keeping with previous sequencing analyses [24] and confirms the observation that interruptions tend to cluster at the 3′ ends of the repeat tract and affect the last 10–15 triplets [22].

To find out whether GAA interruptions play a role in modifying the disease phenotype we examined characteristics of the interruption subgroups comprising the cohort. The subgroups with either 3′ or both 5′ and 3′ interruptions had significantly later ages at disease onset when compared to the group lacking interruptions. The 3′ interruption subgroup also had significantly smaller GAA1 repeat sizes, presumably driving the observed increase in age at disease onset. Although a causal relationship cannot be guaranteed, this finding is in keeping with the well-established observation that those with larger GAA1 repeat sizes have an earlier age at disease onset [2,6,11,24].

The age at disease onset was modelled here as exponentially decreasing with an increasing length of GAA1 repeat size. Similar models have previously been used in other repeat disorders [27,28,29,30,31]. A quadratic model has been used to model the age at disease onset in Friedreich’s ataxia [6,24]. Such a model can be viewed as a second order Taylor approximation of the exponential model used here. However, such an approximation is only valid for comparatively small repeat sizes and will therefore predict clinically unobserved increasing age at disease onset for larger repeat lengths. This is illustrated in Figure S1 where we use a quadratic model to describe the dependence of the age at disease onset on GAA1 repeat size for the present cohort. It can be seen, in particular for the 3′ interruption subgroup, that the predicted age at disease onset increases once a sufficiently high GAA1 repeat size is reached, the inflection point being 1029 GAA1 repeats for the 3′ interruption subgroup (Figure S1D). These erroneous predictions are addressed by the exponential modelling presented here, which would ultimately plateau with a prediction of disease onset occurring at birth for sufficiently high numbers of repeats. An exponential model has previously been used in Huntington’s disease, with an additional offset included to account for the plateau age at disease onset being potentially later in life [31]. However, these models have not stratified patients based on interruption type, as done for the first time in this work.

**Figure 6:**
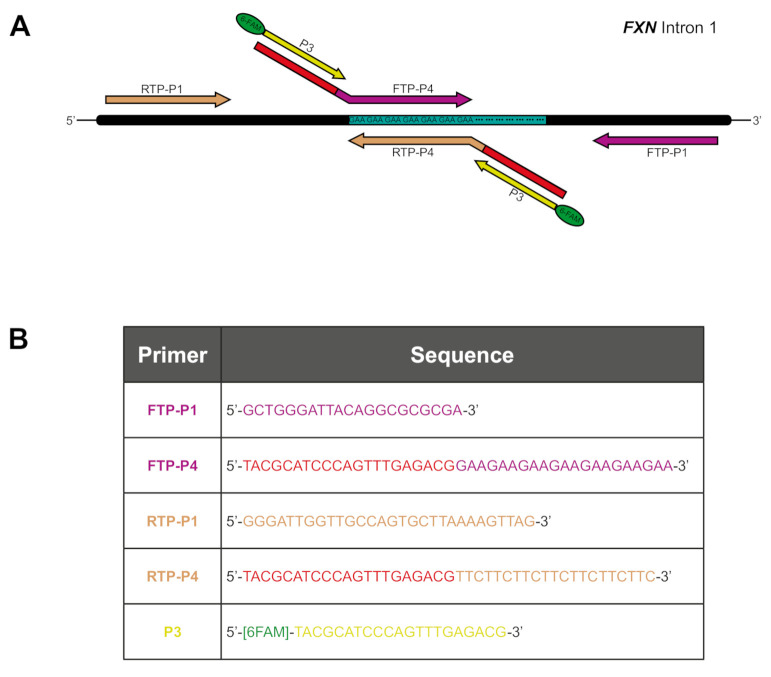
Triplet repeat primed PCR (TP PCR) strategy. (**A**) Schematic of the primer combinations used for Forward (FTP) and Reverse (RTP) TP PCRs with the FTP primers shown in magenta and RTP shown in taupe. The P4 primer tails are shown in red with the 6-FAM-labelled tail-specific primer P3 shown in yellow. (**B**) Table of primer sequences.

While there are undoubtedly many factors dictating the age at disease onset, our findings indicate the importance of stratifying the FRDA cohort based on interruption type in order to obtain more accurate predictions of the age at disease onset. Not accounting for the interruption type, using the model parameters derived from the subgroup lacking interruptions can lead to the predicted age at disease onset being earlier than clinically observed. In particular for the 3′ interruption subgroup, the predicted age at disease onset was significantly lower than that observed clinically (both in terms of age at onset ratio and differences), leading to a median underestimation of the age at disease onset of 8.5 years. Whereas there was no significant difference in prediction accuracy when the group-specific models were used (Figure 6). 

Here we have explored the relationship between the shorter repeat allele (GAA1) and the age at disease onset. However it has previously been observed that there is also an inverse relationship, although much weaker, between the larger repeat allele (GAA2) and the age at disease onset [2,6,11]. There was no significant difference between the GAA2 repeat sizes of the interruption subgroups of our cohort (Figure S3). Indeed, the relationship between GAA2 and age at disease onset is weaker than that for GAA1, with the exponential decay model having an adjusted *R*^2^ of 0.153 (Figure S4) and the quadratic model having an adjusted *R*^2^ of 0.227 (Figure S5). The quadratic model is particularly poor with the subgroup lacking interruptions having a maximal age at disease onset (Figure S5A,B), while the subgroups with interruptions show delayed age at onset for the longest GAA2 repeat lengths (Figure S5A,C–E). To determine which allele is interrupted, we have analysed purified GAA1 and GAA2 alleles from a subset of the cohort by TP PCR and have found that the interruptions are present in the smaller GAA1 allele (data not shown).

The model we have used here shows the significant impact 3′ interruptions have on delaying the age at disease onset in the FRDA patients of our cohort. We have previously shown that interruptions in SCA1 pathogenic CAG repeat tracts also delay the age at disease onset [21]. Meanwhile, loss of the CAA interruption at the 3′ end of the Huntington’s disease CAG repeat tract leads to an earlier age at onset and increased instability of the repeat, whilst CAACAG duplication delays age at onset [32,33]. There could be several explanations for the impact of a 3′ interruption in the GAA repeat tract of *FXN*. Interruptions introduce base mismatches which could inhibit the formation of sticky DNA secondary structures and would alleviate transcription inhibition [22]. It has been proposed that GAA repeat expansion occurs due to template switching during replication, when a leading strand DNA polymerase accidentally switches its template to continue DNA synthesis along the nascent lagging strand [34,35]. It is possible that 3′ interruptions act as an anchor during replication, reducing the number of template-switching events and in turn slowing down the repeat expansion rate. This may also explain the increasing occurrence of individuals with smaller GAA1 repeats in the 3′ interruption subgroup.

The main limitation of using TP PCR to detect small interruptions is its diminishing sensitivity further into the repeat tract, i.e., for larger PCR products. FTP has a detection limit of about 100 repeats in from the 3′ end of the repeat tract. For RTP, the detection limit is about 60 repeats in from the 5′ end of the repeat tract due to the reduced signal in the trace probably caused by the presence of a 16 nucleotide polyA stretch adjacent to the repeat tract. This means that small interruptions, not detected by *Mbo*II digestion, located further into the GAA repeat may be missed. This is why we refer here to the subgroup as lacking 5′ and 3′ interruptions rather than uninterrupted or pure GAA repeats as we cannot be certain that this is not the case.

Sanger sequencing of the interruptions at the 5′ has been hampered by the presence of a 16 nucleotide polyA stretch immediately 5′ to the GAA repeat tract. Sequencing of some of the 3′ interrupted FTP products has revealed single nucleotide insertions or deletions of A or G nucleotides (data not shown). Novel long-read sequencing techniques, such as single-molecule real-time (SMRT) sequencing (Pacific Biosciences) will permit direct sequencing of the entire GAA repeat tract to identify both short and long sequence variation. The ability to identify interruptions both large and small will allow for the further stratification of FRDA cohorts and potential improvement of the models for predicting age at disease onset. This would facilitate further understanding of the role of GAA repeat interruptions in the FRDA phenotype.

## 4. Materials and Methods

### 4.1. Patient Cohort and Ethical Statement

A total of 101 peripheral blood genomic DNA samples were obtained from FRDA patients that had previously undergone GAA repeat expansion size determination. Ethical approval was obtained within the European Union Seventh Framework Programme (FP7/2007–2013) under grant agreement number 242193/EFACTS and from the London—Queen Square Research Ethics Committee (reference 09/H0716/53).

### 4.2. Triplet Repeat Primed PCR (TP PCR)

TP PCR was used to examine the 5′ and 3′ ends of the *FXN* GAA repeat tract independently, with a Reverse TP PCR (RTP) or Forward TP PCR (FTP) assay, respectively. TP PCR was performed using AmpliTaq Gold 360 Master Mix (Applied Biosystems, Waltham, MA, USA) with 400 ng genomic DNA per reaction. FTP primers were adapted from those previously described [25], with the tail-specific primer P3 being 6-FAM-labelled and common between FTP and RTP assays. Figure 6A shows a schematic of the primer binding locations across the *FXN* intron 1 region whilst the table in Figure 6B details the primer sequences. The 20 µL TP PCR reactions contained 1 µL 10 µM P1, 1 µL of a primer mix (10 µM P3; 1 µM P4) and either 2 µL GC enhancer (Applied Biosystems) (for FTP) or a final concentration of 0.85 M betaine (Sigma-Aldrich, Dorset, UK) (for RTP). The following thermocycling conditions were used: 95 °C for 10 min; 35 cycles of 95 °C for 1 min, 52.2 °C for 1 min, 68 °C for 1 min; final extension of 72 °C for 7 min. A total of 2 µL of TP PCR products were then analysed by capillary electrophoresis with 12 µL HiDi Formamide (Applied Biosystems) and 0.3 µL GeneScan 500 LIZ^®^ Size Standard (Applied Biosystems) and separated on an ABI 3730*xl* DNA Analyzer (Applied Biosystems). The resulting output was analysed using GeneMapper software (version 5.0, Applied Biosystems).

### 4.3. Assessing and Modelling the Impact of Interruptions

Non-parametric Kruskal–Wallis and subsequent Dunn’s multiple comparisons tests were performed using Prism statistical software (version 9.1.0, GraphPad Software, San Diego, CA, USA) to test for differences in the age at disease onset and GAA1 repeat sizes between the group lacking interruptions and the three subgroups defined by their interruption location(s).

The observed biological disease process, whereby increased GAA1 repeat size leads to an ever-decreasing reduction in the age at disease onset, was modelled by a group-specific exponential decay according to Equation (1):

(1) $$Age=A_{i}\;e^{-k_{i}.GAA1}$$

Here i indexes the subgroups, i.e., lacking 5′ and 3′ interruptions, only a 5′ interruption, only a 3′ interruption, or both 5′ and 3′ interruption. $A_{i}$ is the age at disease onset, for group i, prior to modulation via GAA1 repeat size. These ages at disease onset exponentially reduce with a per-group rate constant $k_{i}$. The values of $A_{i}$ and $k_{i}$ for each group are shown in Table 1. Both the ages at disease onset and the rate constants are modelled as perturbations about those values for the group lacking interruptions. The eight model parameters were concurrently estimated via log-linear least squares regression of the entire dataset using MATLAB (version R2021a, MathWorks, Inc., Natick, MA, USA).

To assess the impact of having group-specific models, the age at disease onset was calculated for each patient using the model parameters determined for the subgroup lacking interruptions. The age at onset ratio, defined as the observed age at disease onset relative to the predicted age at disease onset was computed, as was the difference between the clinically observed and predicted ages at disease onset. Non-parametric Kruskal–Wallis and subsequent Dunn’s multiple comparisons tests were then performed to test whether these measures differed between those lacking interruptions and each of the subgroups with interruptions.

## 5. Conclusions

This study shows that TP PCR can be used to quickly and easily screen for small interruptions towards the ends of the GAA repeat tract. In our cohort we have observed that individuals with an interruption at the 3′ end of the GAA repeat tract have shorter GAA1 repeat sizes and later ages at disease onset. An exponential decay model can describe the impact of the length of the GAA1 repeat tract on the age at disease onset. Doing so in a group-specific manner, stratifying based on the location of interruptions within the GAA repeat tract, improves the accuracy of the predicted age at disease onset, particularly for the subgroup with 3′ interruptions. To our knowledge, this is the first time an exponential model has been used to describe the relationship between GAA1 repeat size and age at disease onset in an interruption-stratified manner. The evidence presented here reinforces the need to account for the presence, and location, of interruptions in the GAA1 repeat tract and point to 3′ interruptions being a significant disease modifier of the Friedreich’s ataxia phenotype. Given the impact these findings have on the prognosis for patients, they are likely to enrich their genetic counselling. Stratification of patients based on interruption type and location could also benefit intervention therapeutic trials.

## Figures and Tables

**Table 1:** Summary statistics of the cohort used in this study. Data are median (interquartile range). The modelling coefficient ($A_{i}$) and rate constant ($k_{i}$) for each subgroup are also shown.

	Lacking 5′ and 3′ Interruption (*n* = 29)	5′ Interruption (*n* = 19)	3′ Interruption (*n* = 32)	5′ and 3′ Interruption (*n* = 21)
**Age at Onset** (years)	10 (7–14.0)	16 (10–25)	20 (13–30)	16 (7–28)
Number of *FXN* GAA repeats				
**Shorter Allele** (GAA1)	782 (656–960)	683 (483–765)	552 (316–758)	696 (349–827)
**Longer Allele** (GAA2)	1000 (842–1144)	1040 (800–1100)	974 (765–1040)	900 (783–1301)
$A_{i}$	24.5	22.9	48.1	25.3
$k_{i}$	$1.2\times 10^{-3}$	$7\times 10^{-4}$	$1.7\times 10^{-3}$	$9\times 10^{-4}$

## Data Availability

The data presented in this study are available from the corresponding author upon reasonable request.

## Supplementary Materials

The following are available online at https://www.mdpi.com/article/10.3390/ijms22147507/s1, Figure S1: Ages at onset with respect to the smaller FXN GAA (GAA1) repeat size (quadratic dependence), Figure S2: Predicted versus actual age at disease onset for a given number of GAA1 repeats (quadratic dependence), Figure S3: The larger FXN GAA repeat allele (GAA2) sizes do not significantly differ between interruption groups, Figure S4: Ages at onset with respect to the larger FXN GAA (GAA2) repeat size (exponential decay), Figure S5: Ages at onset with respect to the larger FXN GAA (GAA2) repeat size (quadratic dependence).

## References

[1] Winter R.M., Harding A.E., Baraitser M., Bravery M.B. (2008). Intrafamilial correlation in Friedreich’s ataxia. Clin. Genet..

[2] Reetz K., Dogan I., Costa A.S., Dafotakis M., Fedosov K., Giunti P., Parkinson M.H., Sweeney M.G., Mariotti C., Panzeri M. (2015). Biological and clinical characteristics of the European Friedreich’s Ataxia Consortium for Translational Studies (EFACTS) cohort: A cross-sectional analysis of baseline data. Lancet Neurol..

[3] Harding A.E. (1981). Friedreich’s ataxia: A clinical and genetic study of 90 families with an analysis of early diagnostic criteria and intrafamilial clustering of clinical features. Brain.

[4] Hewer R.L., Robinson N. (1968). Diabetes mellitus in Friedreich’s ataxia. J. Neurol. Neurosurg. Psychiatry.

[5] Thoren C. (1962). Diabetes mellitus in Friedreich’s ataxia. Acta Paediatr. Suppl..

[6] Dürr A., Cossee M., Agid Y., Campuzano V., Mignard C., Penet C., Mandel J.-L., Brice A., Koenig M. (1996). Clinical and Genetic Abnormalities in Patients with Friedreich’s Ataxia. N. Engl. J. Med..

[7] Cossée M., Schmitt M., Campuzano V., Reutenauer L., Moutou C., Mandel J.-L., Koenig M. (1997). Evolution of the Friedreich’s ataxia trinucleotide repeat expansion: Founder effect and premutations. Proc. Natl. Acad. Sci. USA.

[8] Montermini L., Andermann E., Labuda M., Richter A., Pandolfo M., Cavalcanti F., Pianese L., Iodice L., Farina G., Monticelli A. (1997). The Friedreich ataxia GAA triplet repeat: Premutation and normal alleles. Hum. Mol. Genet..

[9] Sharma R., De Biase I., Gómez M., Delatycki M.B., Ashizawa T., Bidichandani S.I. (2004). Friedreich ataxia in carriers of unstable borderline GAA triplet-repeat alleles. Ann. Neurol..

[10] Campuzano V., Montermini L., Moltò M.D., Pianese L., Cossée M., Cavalcanti F., Monros E., Rodius F., Duclos F., Monticelli A. (1996). Friedreich’s Ataxia: Autosomal Recessive Disease Caused by an Intronic GAA Triplet Repeat Expansion. Science.

[11] Filla A., de Michele G., Cavalcanti F., Pianese L., Monticelli A., Campanella G., Cocozza S. (1996). The Relationship between Trinucleotide (GAA) Repeat Length and Clinical Features in Friedreich Ataxia. Am. J. Hum. Genet..

[12] Epplen C., Frank G., Miterski B., Santos E.J.M. (1997). Differential stability of the (GAA) n tract in the Friedreich ataxia (STM7) gene. Qual. Life Res..

[13] Tai G., Yiu E.M., Corben L.A., Delatycki M.B. (2015). A longitudinal study of the Friedreich Ataxia Impact Scale. J. Neurol. Sci..

[14] Monrós E., Molto M.D., Martínez F., Canizares J., Blanca J., Vílchez J.J., Prieto F., de Frutos R., Palau F. (1997). Phenotype Correlation and Intergenerational Dynamics of the Friedreich Ataxia GAA Trinucleotide Repeat. Am. J. Hum. Genet..

[15] Galea C.A., Huq A., Lockhart P., Tai G., Corben L.A., Yiu E.M., Gurrin L.C., Lynch D.R., Gelbard S., Durr A. (2016). Compound heterozygousFXNmutations and clinical outcome in friedreich ataxia. Ann. Neurol..

[16] Ohshima K., Sakamoto N., Labuda M., Poirier J., Moseley M.L., Montermini L., Ranum L.P.W., Wells R.D., Pandolfo M. (1999). A nonpathogenic GAAGGA repeat in the Friedreich gene: Implications for pathogenesis. Neurology.

[17] Moseley M.L., Benzow K.A., Schut L.J., Bird T.D., Gomez C.M., Barkhaus P.E., Blindauer K.A., Labuda M., Pandolfo M., Koob M.D. (1998). Incidence of dominant spinocerebellar and Friedreich triplet repeats among 361 ataxia families. Neurology.

[18] McDaniel D.O., Keats B., Vedanarayanan V., Subramony S. (2001). Sequence variation in GAA repeat expansions may cause differential penotype display in Friedreich’s ataxia. Mov. Disord..

[19] Stolle C.A., Frackelton E.C., McCallum J., Farmer J.M., Tsou A., Wilson R.B., Lynch D.R. (2008). Novel, complex interruptions of the GAA repeat in small, expanded alleles of two affected siblings with late-onset Friedreich ataxia. Mov. Disord..

[20] Matsuura T., Fang P., Pearson C.E., Jayakar P., Ashizawa T., Roa B.B., Nelson D.L. (2006). Interruptions in the Expanded ATTCT Repeat of Spinocerebellar Ataxia Type 10: Repeat Purity as a Disease Modifier?. Am. J. Hum. Genet..

[21] Menon R.P., Nethisinghe S., Faggiano S., Vannocci T., Rezaei H., Pemble S., Sweeney M.G., Wood N.W., Davis M.B., Pastore A. (2013). The Role of Interruptions in polyQ in the Pathology of SCA1. PLoS Genet..

[22] Sakamoto N., Larson J.E., Iyer R.R., Montermini L., Pandolfo M., Wells R.D. (2001). GGA·TCC-interrupted Triplets in Long GAA·TTC Repeats Inhibit the Formation of Triplex and Sticky DNA Structures, Alleviate Transcription Inhibition, and Reduce Genetic Instabilities. J. Biol. Chem..

[23] Holloway T.P., Rowley S.M., Delatycki M.B., Sarsero J.P. (2011). Detection of interruptions in the GAA trinucleotide repeat expansion in the FXN gene of Friedreich ataxia. BioTechniques.

[24] Al-Mahdawi S., Ging H., Bayot A., Cavalcanti F., La Cognata V., Cavallaro S., Giunti P., Pook M.A. (2018). Large Interruptions of GAA Repeat Expansion Mutations in Friedreich Ataxia Are Very Rare. Front. Cell. Neurosci..

[25] Ciotti P., Di Maria E., Bellone E., Ajmar F., Mandich P. (2004). Triplet Repeat Primed PCR (TP PCR) in Molecular Diagnostic Testing for Friedreich Ataxia. J. Mol. Diagn..

[26] Warner J.P., Barron L.H., Goudie D., Kelly K., Dow D., Fitzpatrick D.R., Brock D.J. (1996). A general method for the detection of large CAG repeat expansions by fluorescent PCR. J. Med. Genet..

[27] Ranum L.P.W., Chung M.-Y., Banfi S., Bryer A., Schut L.J., Ramesar R., Duvick L.A., McCall A., Subramony S.H., Goldfarb L. (1994). Molecular and Clinical Correlations in Spinocerebellar Ataxia Type I: Evidence for Familial Effects on the Age at Onset. Am. J. Hum. Genet..

[28] Goldfarb L.G., Vasconcelos O., Platonov F.A., Lunkes A., Kipnis V., Kononova S., Chabrashvili T., Vladimirtsev V.A., Alexeev V.P., Gajdusek D.C. (1996). Unstable triplet repeat and phenotypic variability of spinocerebellar ataxia type 1. Ann. Neurol..

[29] Pulst S.-M., Nechiporuk A., Nechiporuk T., Gispert S., Chen X.-N., Lopes-Cendes I., Pearlman S., Starkman S., Orozco-Diaz G., Lunkes A. (1996). Moderate expansion of a normally biallelic trinucleotide repeat in spinocerebellar ataxia type 2. Nat. Genet..

[30] Van De Warrenburg B.P.C., Hendriks H., Dürr A., Van Zuijlen M.C.A., Stevanin G., Camuzat A., Sinke R.J., Brice A., Kremer B.P.H. (2005). Age at onset variance analysis in spinocerebellar ataxias: A study in a Dutch-French cohort. Ann. Neurol..

[31] Langbehn D.R., Brinkman R.R., Falush D., Paulsen J., Hayden M.R., On behalf of an International Huntington’s Disease Collaborative Group (2004). A new model for prediction of the age of onset and penetrance for Huntington’s disease based on CAG length. Clin. Genet..

[32] Genetic Modifiers of Huntington’s Disease (GeM-HD) Consortium (2019). CAG Repeat Not Polyglutamine Length Determines Timing of Huntington’s Disease Onset. Cell.

[33] Wright G.E., Collins J.A., Kay C., McDonald C., Dolzhenko E., Xia Q., Bečanović K., Drögemöller B.I., Semaka A., Nguyen C.M. (2019). Length of Uninterrupted CAG, Independent of Polyglutamine Size, Results in Increased Somatic Instability, Hastening Onset of Huntington Disease. Am. J. Hum. Genet..

[34] Shishkin A.A., Voineagu I., Matera R., Cherng N., Chernet B.T., Krasilnikova M.M., Narayanan V., Lobachev K.S., Mirkin S.M. (2009). Large-Scale Expansions of Friedreich’s Ataxia GAA Repeats in Yeast. Mol. Cell.

[35] Gerhardt J., Bhalla A.D., Butler J.S., Puckett J.W., Dervan P.B., Rosenwaks Z., Napierala M. (2016). Stalled DNA Replication Forks at the Endogenous GAA Repeats Drive Repeat Expansion in Friedreich’s Ataxia Cells. Cell Rep..

