# Supplementary material for: Interruptions of the FXN GAA Repeat Tract Delay the Age at Onset of Friedreich’s Ataxia in a Location Dependent Manner
Source: Int J Mol Sci. 2021 Jul 13;22(14):7507. doi: 10.3390/ijms22147507 (PMC8307455; doi:10.3390/ijms22147507)
Supplement: Supplementary file 1 [file ijms-22-07507-s001.zip › ijms-1246011_Supplementary-Material.pdf]

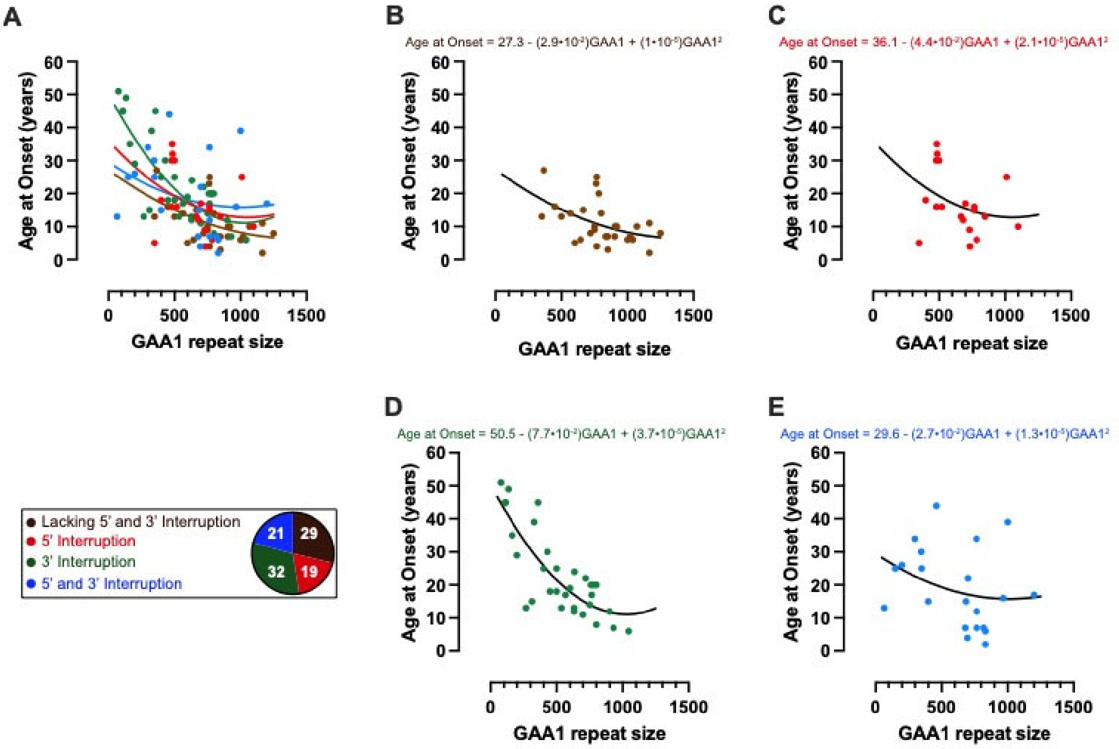

Figure S1 – Ages at onset with respect to the smaller *FXN* GAA (GAA1) repeat size. The dependence of the age at disease onset on GAA1 repeat size has previously been modelled as a quadratic dependence, which is done here for comparison (adjusted $R^2 = 0.429$; F-statistic 15.3, $P = 2.44 \times 10^{-9}$). **(A)** Actual and modelled dependence of the age at disease onset on GAA1 repeat size for the whole FRDA cohort (n = 101). The group membership is colour-coded according to the legend, which also indicates the number of patients per group. **(B-E)** show the data, model results and model equation for each group separately. This model predicts an infeasible rise in age at disease onset at large repeat size in the subgroups with interruptions (C-E).

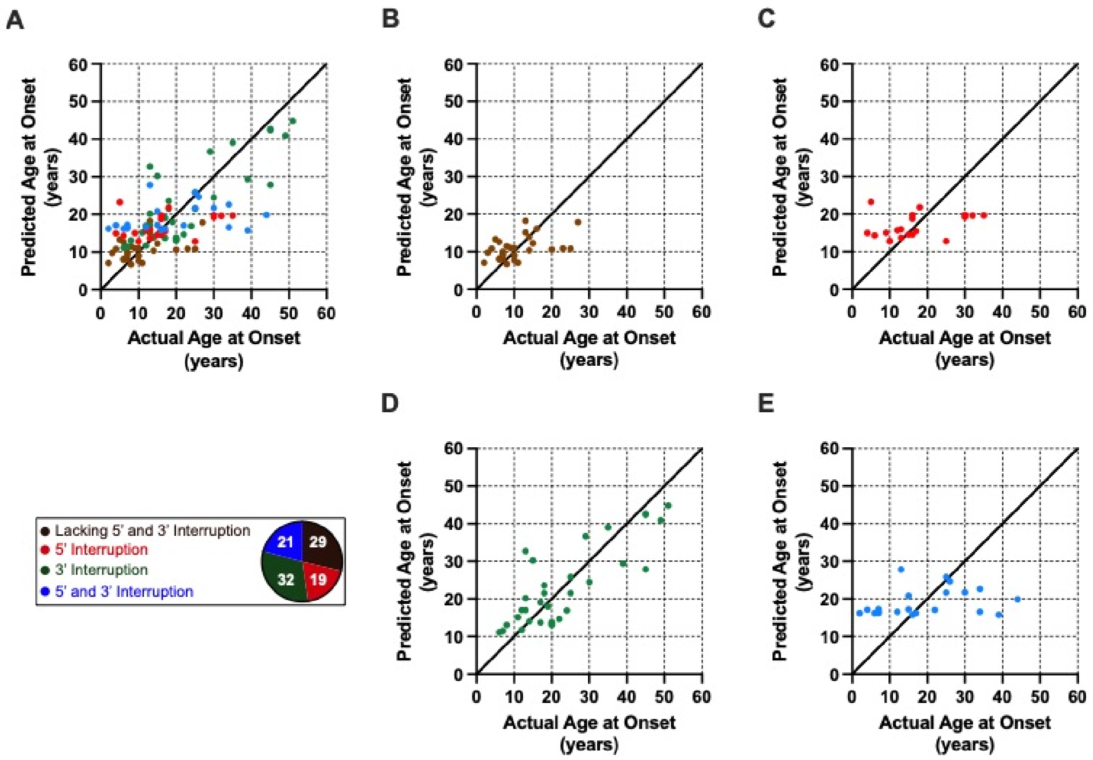

Figure S2 – Predicted versus actual age at disease onset for a given number of GAA1 repeats. (A) Actual and predicted ages at disease onset for the whole FRDA cohort (*n* = 101) based on the quadratic model. The subgroup membership is colour-coded according to the legend, which also indicates the number of patients per subgroup. **(B-E)** show the predicted versus actual age at disease onset for each subgroup separately. In each graph, the solid black line indicates identical predicted and actual ages at disease onset.

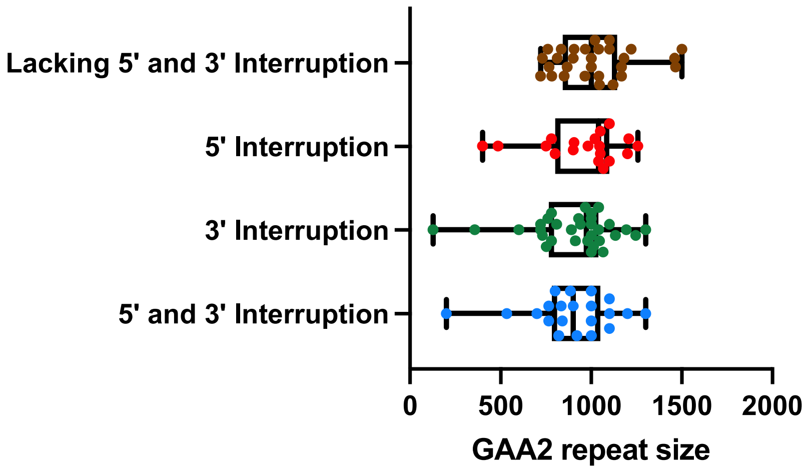

Figure S3 – The larger *FXN* GAA repeat allele (GAA2) sizes do not significantly differ between interruption subgroups. Box-and-whisker plot showing the distribution of the GAA2 repeat sizes for each interruption subgroup of the cohort. Kruskal-Wallis and subsequent Dunn's multiple comparisons tests identified no significant differences between the subgroups.

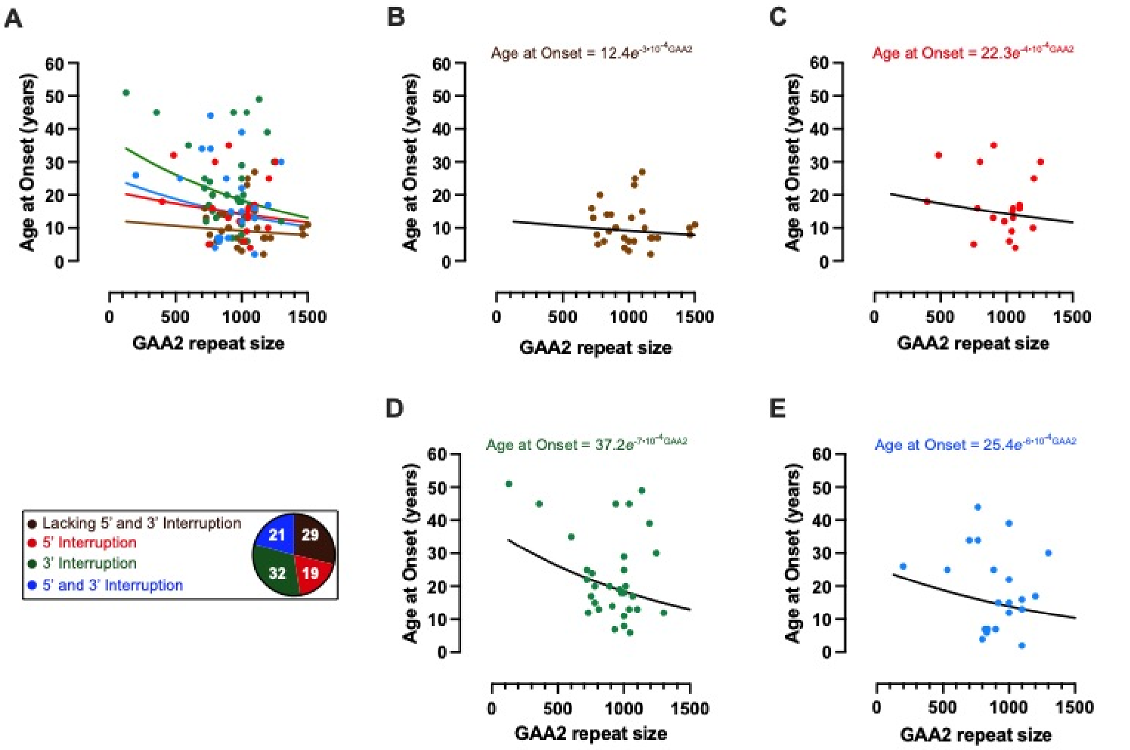

Figure S4 – Ages at onset with respect to the larger *FXN* GAA (GAA2) repeat size. The relationship between the age at disease onset and GAA2 repeat size was modelled as an exponential decrease, on a groupwise basis (adjusted R^2 = 0.153; F-statistic 3.58, P = 1.86×10^{-3}). **(A)** Actual and modelled dependence of the age at disease onset on GAA2 repeat size for the whole FRDA cohort (n = 101). The group membership is colour-coded according to the legend, which also indicates the number of patients per group. **(B-E)** show the data, model results and model equation for each group separately.

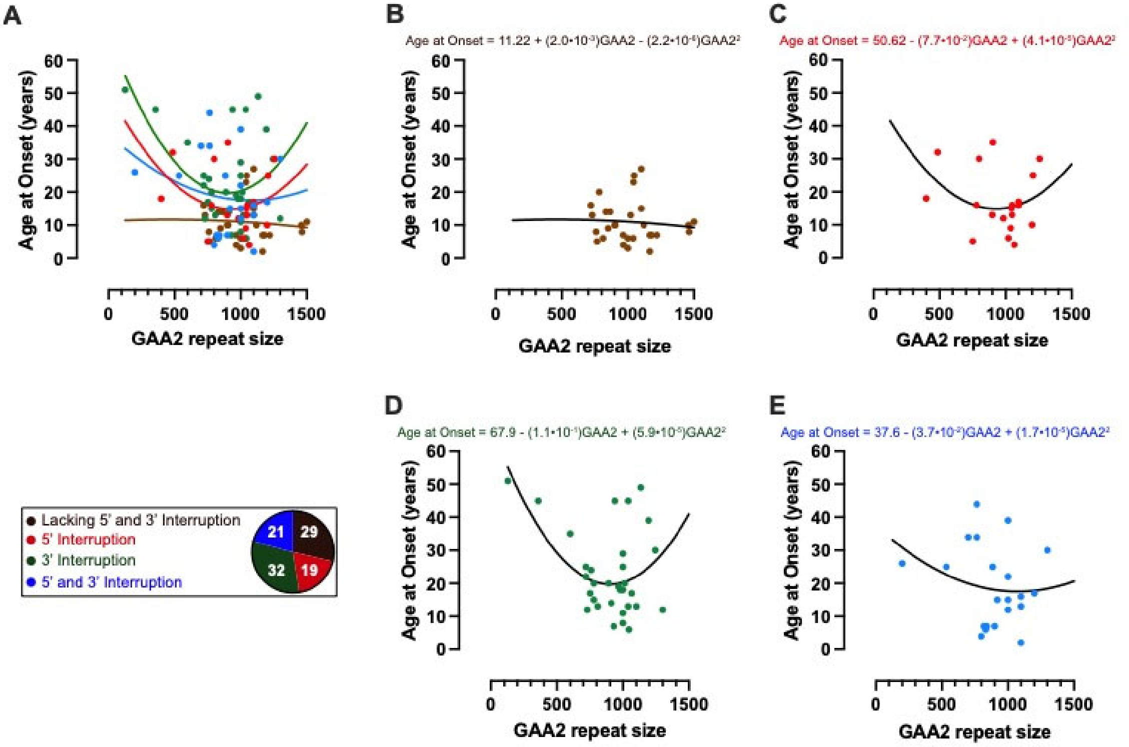

Figure S5 – Ages at onset with respect to the larger *FXN* GAA (GAA2) repeat size. The dependence of the age at disease onset on GAA repeat size has previously been modelled as a quadratic dependence, which is done here for comparison (adjusted R^2 = 0.227; F-statistic 3.67, $P = 2.54\times10^{-4}$). **(A)** Actual and modelled dependence of the age at disease onset on GAA2 repeat size for the whole FRDA cohort (*n* = 101). The group membership is colour-coded according to the legend, which also indicates the number of patients per group. **(B-E)** show the data, model results and model equation for each group separately. The modelling results are infeasible: the quadratic term is negative for the subgroup lacking interruptions leading to a maximal age at disease onset for intermediate GAA2 repeat size (A, B), while the subgroups with interruptions show increasing age at disease onset for the longest GAA2 repeat lengths (A, C-E).
